# Exploration of verbal repetition in people with dementia using an online symptom-tracking tool

**DOI:** 10.1017/S1041610216002180

**Published:** 2017-03-09

**Authors:** Emily Reeve, Pierre Molin, Amaris Hui, Kenneth Rockwood

**Affiliations:** 1 Geriatric Medicine Research Unit, Dalhousie University and Capital Health, Halifax, Nova Scotia, Canada; 2 Cognitive Decline Partnership Centre, Kolling Institute of Medical Research, Sydney Medical School, The University of Sydney, New South Wales, Australia; 3 Division of Geriatric Medicine, Dalhousie University, Halifax, Nova Scotia, Canada; 4 Division de gériatrie, Université Laval, Québec, Canada; 5 DGI Clinical Inc., Halifax, Nova Scotia, Canada

**Keywords:** Alzheimer disease, dementia, Internet, caregiver, verbal repetition

## Abstract

**Background:**

Online tools can be used by people with dementia and their caregivers to self-identify and track troubling symptoms, such as verbal repetition. We aimed to explore verbal repetition behaviors in people with dementia.

**Methods:**

Participants were recruited via an online resource for people with dementia and their caregivers. Respondents were instructed to complete information about symptoms that are most important to them for tracking over time. In this cross-sectional study, we analyzed data pertaining to individuals with dementia who had at least three symptoms selected for tracking.

**Results:**

Of the 3,573 participants who began a user profile, 1,707 fulfilled criteria for analysis. Verbal repetition was identified as a treatment target in 807 respondents (47.3%). Verbal repetition was more frequent in individuals with mild dementia compared to those with moderate and severe dementia (57.2% vs. 36.0% and 39.9%, p < 0.01) and in those with Alzheimer's disease versus other dementias (65.2% vs. 29.7%, p < 0.001). Repetitive questioning was the most frequent type of verbal repetition (90.5% of individuals with verbal repetition). Verbal repetition was most strongly associated with difficulties operating gadgets/appliances (OR 3.65, 95%CI: 2.82–4.72), lack of interest and/or initiative (3.52: 2.84–4.36), misplacing or losing objects (3.25: 2.64–4.01), and lack of attention and/or concentration (2.62: 2.12–3.26).

**Conclusions:**

Verbal repetition is a common symptom in people at all stages of dementia but is most commonly targeted for monitoring and treatment effects in its mild stage. Much research is required to further elucidate the underlying mechanisms and the effect of different treatment strategies.

## Introduction

The clinical expression of disease in dementia varies both within and between individuals. Age, cultural background, co-morbidities, cognitive reserve, compensatory changes, neuropathological features, and disease stage all contribute to the intrinsic heterogeneity of dementia (Cohen-Mansfield, 2000; Rockwood, 2010). Because disease manifestations vary immensely between patients, dementia management needs to take into account the individual and caregiver's dementia experience. The inherent clinical meaningfulness of many standardized tests widely used in memory clinics (e.g. Montreal Cognitive Assessment (MoCA) and Mini-Mental State Examination (MMSE)) may be important to healthcare providers for diagnosis and management but may not coincide with what caregivers consider to be “clinically meaningful” as these tests often do not target symptoms of greatest burden or distress (Rockwood, 2010; Shabbir and Sanders, 2014).

Studying heterogeneity in symptoms and presentation of dementia and the response to treatment can lead to a better appreciation of brain functioning. Additionally, study of individual symptom types and a focus on individual goal setting and attainment can enhance patient-centered care. To this end, we have structured aspects of collateral history by employing the SymptomGuide^™^ (SG), a standardized dementia symptom inventory that can be used by caregivers to track symptoms of most importance to them (Rockwood, 2010). This is one way in which online tools offer new approaches for understanding individuals’ experiences of dementia (McKechnie *et al.*, 2014; Cristancho-Lacroix *et al.*, 2015). Here, we used SG data to study verbal repetition, a commonly targeted yet poorly studied symptom of dementia.

Verbal repetition in people with dementia is one important manifestation of reduced cognition. It can occur early in dementia and is amongst the behaviors that most trouble caregivers (Hwang *et al.*, 2000; Ready *et al.*, 2003; Rockwood *et al.*, 2014). Repetitive verbalizations can take the form of repetitive questions, story-telling, statements, and talk on a topic and repeating words (Cook *et al.*, 2009). Like other symptoms in dementia, verbal repetition behaviors can fluctuate and have been reported as a marker of a “bad day,” as described by caregivers (Rockwood *et al.*, 2014). Even so, little is known of how verbal repetition manifests in the daily lives of people with dementia.

The purpose of this study was to characterize verbal repetition in a large population of people with dementia online using a symptom-tracking website. The specific objectives were to determine how often and at which stage of dementia verbal repetition was reported as a symptom of interest for tracking disease progression. In addition, we examined how it related to other reported symptoms.

## Methods

### Design, participants, and instrument

Data for this cross-sectional study were obtained from an online questionnaire completed by informal caregivers providing assistance to community-dwelling care recipients with cognitive impairment. Caregivers were spouses, children, or other care providers. The participants were recruited online at www.DementiaGuide.com. This website is a reference for people to learn about Alzheimer's disease and dementia and, in particular, the symptoms of dementia. Additionally, it provides a function for users to create a symptom profile, enabling them to track the changes in symptoms over time; this is called the SymptomGuide^™^ (SG) (Rockwood, 2010). People who access the SG can either subscribe or complete a questionnaire for free access. The SG provides a standardized dementia symptom inventory of 60 symptoms, including verbal repetition, and aims to represent all stages of cognitive impairment. Information is provided about each symptom, such as the stage at which it most commonly occurs and typical management strategies. For each symptom, about a dozen descriptors are present, which provide a menu for selection; users can also write in their own description if they choose. Users are instructed to select the symptoms that are most relevant to them to track the course of dementia and/or the effect of treatment. That is, not all symptoms that may be present in the person with dementia will be selected, but instead, only those viewed by the user to be a symptom of interest to be tracked over time. Users are asked to indicate the frequency of each symptom. They can also rank the symptoms as most to least important to them. Respondents were also able to report a diagnosis of dementia when present and give information about current medications.

The content of DementiaGuide has been independently judged as credible (high quality and comprehensive) (Dillon *et al.*, 2013) and the SG has been validated clinically and against the Dependence Scale (Rockwood *et al.*, 2012). The symptom library in SG contains ten descriptions of verbal repetition behaviors as well as the option to write their own description. These descriptions can be grouped into three categories: repetitive questions, repetitive statements and stories, and verbal perseveration (repetition of the same word or short phrase).

The site has been available since September 2006 and we examined data collected until February 2015. To enable staging of the severity of dementia (see below), only individuals in whom at least three symptoms were reported were included in analysis. For this study, we also excluded people with mild cognitive impairment (i.e. without dementia).

### Statistical analysis

In addition to information provided by respondents, each individual had their dementia classified as mild, moderate, or severe using a staging algorithm. The staging algorithm was developed using an artificial neural network and has been validated for use in SG (details reported in full previously (Rockwood *et al.*, 2013)). The algorithm requires only descriptions of symptoms reported using the SG to assign each individual as having mild, moderate, or severe dementia, although a minimum of three symptoms are required.

Data were summarized using descriptive statistics. Testing for significant differences between groups was done with a Student's t-test for continuous data and Pearson's *χ*^2^ test for categorical data. Statistical software used for the analysis was R v3.02 and a p value of <0.05 was considered significant.

To test for associations between verbal repetition and other symptoms selected by the users in the SG, odds ratios (ORs) and 95% confidence intervals were calculated using IBM SPSS Statistics version 22. To identify symptoms that were co-reported for tracking with the greatest association with verbal repetition in this exploratory work, we reported those in which the 95% confidence interval was greater than, and did not cross 2.00.

### Ethics

The study was approved by the Research Ethics Committee of the Capital District Health Authority, Nova Scotia. SG users agreed to terms of use. Respondents gave informed consent to disclose their answers for the purpose of advancing research on dementia. Users are assured that data provided for research purposes will be presented in an aggregate manner, without any information that could be used to identify individuals or respondents personally.

## Results

### Sample description

Of the 3,573 participants who began a user profile, 2,264 completed both a symptom profile in which they targeted symptoms for tracking and a user profile in which they reported patient and care characteristics. Of these 2,264, 1,707 reported a dementia diagnosis, with at least three target symptoms and thus were included in the analysis. Overall, verbal repetition was the most common of the 60 possible symptoms reported as a target for monitoring, in 807 individuals (47.2%). When identified, verbal repetition was ranked as the most important symptom in 34.1% and was one of the top three symptoms in 65.3% in our respondents. Compared to those in whom it was not targeted (*N* = 900), individuals in whom verbal repetition was targeted were significantly older and more often were women, as well as have differences in living arrangements and use of medications to treat dementia (Table 1).

**Table 1.** Subject characteristics

	verbal	verbal	
	repetition	repetition	
	targeted	not targeted	
	(*n* = 807)	(*n* = 900)	p-value
Age: mean (SD)	78.5 (7.4)	79.3 (8.9)	0.046
Sex: % women (*N*)	66.2 (534)	61.4 (553)	0.048
Education: % (*N*)			0.102
More than high school	29.2 (236)	19.8 (178)	
At most high school	21.1 (170)	20.4 (165)	
Missing	49.7 (401)	61.9 (557)	
Living arrangements: % (*N*)			<0.001
Alone	8.1 (65)	6.4 (58)	
At home with help	57.5 (464)	35.4 (319)	
Retirement/nursing home	6.1 (49)	9.8 (88)	
Missing	28.4 (229)	48.3 (435)	
Treated with a cholinesterase inhibitor: % (*N*)	67.0 (412)	51.8 (335)	<0.001
Missing	6.2 (38)	5.9 (38)	
Type of dementia: % (*N*)			<0.001
Alzheimer	46.2 (373)	22.1 (199)	
Lewy Body	2.0 (16)	4.1 (37)	
Frontotemporal	1.5 (12)	4.3 (39)	
Parkinson	0.1 (1)	1.4 (13)	
Vascular	5.8 (47)	10.1 (91)	
Missing	44.4 (358)	57.9 (521)	

**Figure 1.**
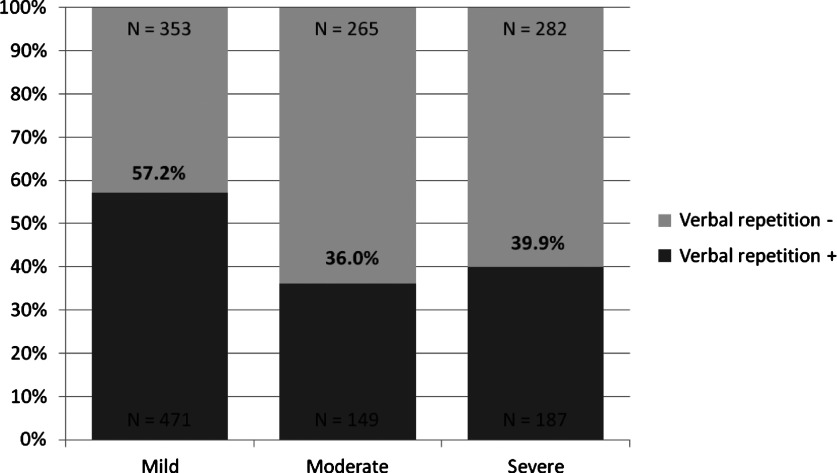
Proportion of patients in whom verbal repetition was identified as a target for tracking, by dementia stage.

The dementia type was specified by users in 828 participants (data missing on 879). For these 828, verbal repetition was more often targeted in people with AD than in those with other dementias (373/572; 65.2%, 76/256; 29.7%, p < 0.001). Verbal repetition was targeted across all stages, although significantly more so in patients with mild dementia than in those in the moderate or severe stages (p < 0.001 for both) (Figure 1). This pattern remained in the subgroup of patients with AD; verbal repetition was targeted in 74.2% of people with mild AD, 45.7% of those with moderate AD, and 47.9% of those with severe AD (p < 0.001).

### Description of the episodes

In individuals who targeted verbal repetition, repetitive questioning was the most common type across all stages (90.8%). Repetitive story/information telling (60.1%) and verbal perseveration/other (23.8%) were less frequent (Table 2). The most common descriptors were asking repeatedly for details of upcoming events or appointments and asking for the time of day or date. The occurrence of repetitive questioning did not differ significantly across the dementia stages (p ≥ 0.066), whereas repetitive stories and information and verbal perseveration were targeted significantly more frequently in individuals with moderate and severe dementia (p < 0.002 mild vs. moderate and mild versus severe for both).

**Table 2.** Types of verbal repetition reported as a symptom to track by stage of dementia (% (*n*) of patients by stage)

	mild	moderate	severe	all patients
verbal repetition description	*n* = 471	*n* = 149	*n* = 187	*n* = 807
**Repetitive questioning**	92.1 (434)	91.3 (136)	87.2 (163)	90.8 (733)
Asks repeatedly about upcoming events or appointments	47.3 (223)	66.4 (99)	60.4 (113)	53.9 (435)
Asks repeatedly about the time of day or date	56.7 (267)	49.0 (73)	39.6 (74)	51.3 (414)
Asks repeatedly about things that have already happened	48.8 (230)	47.7 (71)	48.1 (90)	48.2 (391)
Asks repeatedly about whether something was done	48.6 (229)	41.6 (62)	49.2 (92)	47.5 (383)
Asks repeatedly the same question - other	26.8 (126)	40.3 (60)	41.2 (77)	32.6 (263)
**Repetitive stories/information**	47.6 (224)	73.8 (110)	80.7 (151)	60.1 (485)
Tells a story more than once during a single conversation	33.3 (157)	54.4 (81)	61.5 (115)	43.7 (353)
Tells the same story at successive encounters with others (week/week)	27.6 (130)	52.3 (78)	64.2 (120)	40.6 (328)
Tells stories/information despite been told he/she is repeating	15.7 (74)	34.2 (51)	48.7 (91)	26.8 (216)
Tells new information that they have learned multiple times a day	15.1 (71)	30.2 (45)	31.0 (58)	21.6 (174)
**Verbal perseveration and other**	17.8 (84)	36.2 (54)	28.9 (54)	23.8 (192)
Says the same word/phrase over and over	9.3 (44)	25.5 (38)	24.6 (46)	15.9 (128)
Other descriptor	11.9 (56)	20.8 (31)	11.8 (22)	13.4 (109)

The median frequency of verbal repetition was 5.0 episodes/day (range 0.07–624.0) with no statistical difference based on the stage of dementia in the individual (p = 0.079).

### Associated symptoms

Out of the other symptoms that could be selected in SG for tracking, verbal repetition was most strongly associated with difficulties operating gadgets/appliances (OR 3.65, 95% CI: 2.82–4.72), lack of interest and/or initiative (3.52: 2.84–4.36), misplacing or losing objects (3.25: 2.64–4.01), and lack of attention and/or concentration (2.62: 2.12–3.26).

## Discussion

### Summary of results and comparison with previous literature

We used information collected from an online survey to explore how verbal repetition is experienced in the daily lives of community-dwelling individuals with dementia and their informal caregivers. The proportion of individuals in whom verbal repetition was tracked in our online survey (just under half) is within the range of 31%–90% reported in previous studies (Teri *et al.*, 1992; Hope *et al.*, 1997; Hwang *et al.*, 2000; Cullen *et al.*, 2005). This wide range in prevalence estimates may reflect differences in definitions and timeframe/frequency. For example, Hope *et al.* (1997) inquired about repeated requests or demands only, whereas Terri *et al.* (1992) asked about repeated questions at all in the previous week. Other investigations have relied on formal testing done in non-conversational and non-clinical settings to describe verbal repetition (Bayles *et al.*, 2004), how these studies relate to the patient's and caregiver's real-world experience is unclear. In any case, it is worth underscoring that we did not inquire about prevalence per se – only whether, when present, it was a target for tracking change, e.g. in response to treatment, or across the course of the dementia, or simply as a way for caregivers to share with others who use their personal account (such as family members in other locales) about how the person for whom they were caring was faring.

We have demonstrated that verbal repetition is an important symptom of dementia. When selected for tracking, it was ranked in the top three symptoms two-thirds of the time. This observation indicates that verbal repetition can cause significant burden to caregivers (although not directly measured in this study), and as such it is important to investigate causes and effective management strategies. Unfortunately, even if verbal repetition is frequent and important for patients and their caregivers, few drug trials have measured verbal repetition as an outcome. Even so, a double blind, randomized, placebo controlled study found that verbal repetition does respond to treatment with cholinesterase inhibitors in patients with mild-moderate AD (Rockwood *et al.*, 2007). Verbal repetition was selected for tracking in individuals at all dementia stages in this study but was more commonly selected in people with mild dementia than in those in a moderate or severe stage. Similarly, Hwang *et al.* (2000) found that verbal repetition behaviors were reported early in the disease (within two years of diagnosis) but were not related to age of onset or MMSE. These results could reflect that verbal repetition is more common in the early stages of dementia, or that other symptoms become more concerning as the disease progresses and that verbal repetition becomes less salient.

Our study also found that verbal repetition was more likely to be reported in women and in patients with AD. This is contrary to the study by Hwang *et al.* (2000) who found no relationship to gender or type of dementia but consistent with the one by Cullen *et al.* (2005) whose data suggest that repetitive questions were more common amongst women. Whether this gender effect is related to the dementia process remains unclear. Interestingly, women are more vulnerable to semantic and episodic memory decline in AD and this could contribute to this phenomenon (McPherson *et al.*, 1999). On the other hand, even data on healthy participants suggest that women ask more questions (Fitzpatrick *et al.*, 1995). Thus, if there is a gender difference in repetitive questioning, it could reflect an underlying heterogeneity in the use of linguistic forms in the speech of men and women.

Consistent with previous studies (Hwang *et al.*, 2000; Cullen *et al.*, 2005; Cook *et al.*, 2009), we found that repetitive questioning was the most common type of verbal repetition. Repetitive questioning was targeted as a symptom for tracking at all dementia stages in similar proportions, whereas repetitive story/information telling and verbal perseveration were targeted in a greater proportion of individuals with moderate and severe stages of dementia, versus those with mild.

### Pathophysiological cause of verbal repetition

Our analysis of the symptoms that were most highly associated with repetitive verbalization may provide insight into the underlying pathophysiological cause. Even so, beyond a broadly construed (and therefore less informative) notion of “executive function,” no common theme was identified in the top four symptoms: difficulties operating gadgets/appliances, lack of interest and/or initiative, misplacing or losing objects, and lack of attention and/or concentration. As the majority of respondents completing the online survey are caregivers, we cannot discount the possibility that the symptoms identified as important to the caregiver may be a reflection of caregiver characteristics and not purely due to the symptoms of the individual with dementia.

The associations with lack of attention and difficulties operating gadgets may indicate variants of dysexecutive function, whereas misplacing objects might more strongly suggest memory impairment. However, misplacing objects involves both inability to recall where an item was placed and inappropriate placement of objects which may, again be due to impaired planning or monitoring, as part of a dysexecutive syndrome and not just memory impairment (Hamilton *et al.*, 2009). How verbal repetition arises remains unclear, though studies tend to differentiate repetitive questioning from repetitive storytelling/statements and perseveration. This may be an important differentiation for future studies to tease out the causative mechanisms as our study found that there was a different distribution of targeting repetitive questions versus repetitive statements and stories and verbal perseveration across the different stages of dementia.

Repetitive questioning, commonly about upcoming events, whereabouts of people and objects, and temporal orientation, is believed to be an amnesic behavior. Even though anterograde amnesia is the clinical hallmark of AD, repetitive questioning is not reported in every AD patient. This suggests that other factors influence its occurrence (Ready *et al.*, 2003; Cullen *et al.*, 2005; Asp *et al.*, 2006). A perfusion SPECT scan study in AD patients demonstrated that repetitive questioning positively correlated with greater bilateral cerebral blood flow to the pericallosal regions (Kishimoto *et al.*, 2010). Considering that AD patients with severe memory disturbance tend to show a decrease of cerebral blood flow in this region, this finding too suggests that memory dysfunction alone does not account for repetitive questioning. A separate theory is that repetitive questioning stems from anxiety, agitation, and emotional disturbances and is often accompanied with repetitive verbalizations of health complaints, fears, and concerns (Volicer *et al.*, 2001). Our study found that asking repeatedly for details of upcoming events or appointments was the most frequent manifestations of verbal questions, which lends support to this theory. Or it could reflect a problem conceiving how one action entails the next, that is, impairments in sequencing (executive dysfunction).

Repetitive stories and statements are thought to be due to disturbances in executive functioning. The inability to shift attention away from one topic of conversation to another, impaired response inhibition and poor working memory capacity are specifically believed to be related to these verbal comportments (Perry *et al.*, 2000; Cook *et al.*, 2009; Miozzo *et al.*, 2013). One smaller study (*N* = 54 participants with AD) found an association between higher dysexecutiveness and repetitive statements/stories but not with repetitive questions (Cullen *et al.*, 2005).

In relation to executive functioning, recurrent verbal perseverations occur early in the course of AD and has been held to imply frontal lobe involvement rather than memory dysfunction – as demonstrated by the lack of significant association with general memory measures (Bayles *et al.*, 2004; Possin *et al.*, 2005; Pekkala *et al.*, 2007). At the neuropharmacological level, recurrent perseveration and deficits in attentional switching have been attributed to resistance of catecholaminergic and cholinergic activity in the prefrontal cortex (McNamara and Albert, 2004). It is important to note that our definition of verbal repetition excluded morpheme repetitions where patients have impairment in their ability to activate a target lexical item, which relates to language processing rather than memory or executive functions (Miozzo *et al.*, 2013).

### Strengths, limitations, and future directions

Our approach of using an online resource about dementia for research is innovative and this method allowed us access to caregivers’ views of the symptoms of dementia. The utilization of web-based systems for information sharing, online peer support, risk reduction tools, and strategies and even web-based cognitive training for people with dementia and their caregivers is expanding; however, the internet's potential clinical applications remain relatively untapped by the medical community in caring for people with dementia (McKechnie *et al.*, 2014; Cristancho-Lacroix *et al.*, 2015; Pot *et al.*, 2015). This study adds to previous research (Rockwood *et al.*, 2012; Rockwood *et al.*, 2015), establishing the feasibility of an online tool that is both a resource for consumers and a real world data-source for research. Nonetheless, data collected online need to be utilized with prudence.

Our data should be interpreted with caution. For studies of verbal repetition, our sample size is large but is subjective in being observer reported, including on key features such as diagnosis, symptoms, and frequencies. We did not systematically inquire about whether verbal repetition was present, only if it was targeted as a symptom for tracking over time. In other words, this is not a symptom checklist, but rather a compendium of which symptoms crossed enough of a threshold of importance or concern to be followed as treatment targets. Likewise, we had no information on non-targeted symptoms. Therefore, we were constrained to look at associations but not prevalence estimates. Moreover, some data on subject's characteristics and symptom description were missing, making statistical comparison difficult. Our algorithm to classify patients according to their dementia stage also has limitations. A minimum of three reported symptoms was necessary to use it so that we are not able to comment about associated symptoms when fewer than three were specified.

This was a cross-sectional study; however, data were collected over an 8 year period (only first entries were used). It is possible that alterations in guidelines and advances in the care of people with dementia occurred throughout this time period, which may have impacted users’ responses. However, as the content of the SG and the questions asked remained constant over this period, we believe that our analysis remains valid. Additionally, we did not examine the duration that verbal repetition had been experienced. Future research should investigate the duration of symptoms and how the severity and frequency alter over time, as well as the relationship with patients’ quality of life and caregiver burden to provide important insight into how we can better care for people with dementia and their caregivers.

## Conclusion

Verbal repetition is common in individuals at all stages of dementia but is most frequently identified as a symptom of use to track in individuals with mild dementia. It was selected as a symptom to track in approximately half of all respondents and two-thirds of those with AD. Repetitive questions were the most common type of verbal repetition behaviors and were tracked by similar proportions of users across the stages of dementia. Repetitive story/information telling and verbal perseveration were less common but were targeted in a greater proportion of individuals with moderate and severe stages of dementia. As such, there may be different underlying causes of the different types of verbal repetition. The symptoms seen in association with verbal repetition suggest that reduced executive function and memory impairment play a role in repetitive verbal behaviors. Further research is needed to better understand the basis of this phenomenon and how to best care for people with dementia who experience these symptoms and their caregivers.

## Conflict of interest

KR founded and has shares in DGI Clinical, a company that has contracts with pharma for individualized outcome measurement and advanced data analytics in Alzheimer disease, Parkinson disease, and other disorders. The data for these analyses were supplied by DGI Clinical, from its website.

## Description of author roles

E Reeve was involved in conception of the manuscript, conducted data analysis and interpretation, critically reviewed and revised the draft and prepared it for submission. P Molin formulated the research question, designed the study and drafted the manuscript. A Hui was involved in concept of the manuscript, contributed to the draft and critically review the draft. K Rockwood designed the study, was involved in interpreting the results, and critically reviewed the manuscript.
